# Two suspected sequential retinal arterial macroaneurysms in a single eye with secondary nonvascular cystoid macular edema: 5-year follow-up: a case report

**DOI:** 10.3389/fmed.2026.1953333

**Published:** 2026-10-16

**Authors:** Kaiyao Chi, Daoxi Lei, Tong Guo, Xiaoding Shui, Huili Li

**Affiliations:** 1 Department of Ophthalmology, The First Affiliated Hospital of Chongqing College of Traditional Chinese Medicine, Chongqing, China; 2 Chongqing College of Traditional Chinese Medicine, Chongqing, China

**Keywords:** hypertension, multimodal image, non-vascular cystoid macular edema, retinal arterial macroaneurysm, vitreous hemorrhage

## Abstract

**Background:**

Retinal arterial macroaneurysm (RAM) is an acquired vascular disorder. The long-term natural history of multiple RAMs in a single eye complicated by nonvascular cystoid maculopathy (NVCM) is rarely documented.

**Case presentation:**

A 65-year-old hypertensive woman presented with acute vision loss in her left eye secondary to severe vitreous hemorrhage. She underwent pars plana vitrectomy with intravitreal recombinant tissue plasminogen activator thrombolysis and silicone oil tamponade. Postoperative multimodal imaging supported the diagnosis of two suspected sequential RAMs confined to the left eye. Over a 5-year follow-up, the two lesions exhibited divergent outcomes: one developed fibrosis and persistent NVCM associated with severe initial hemorrhage and inadequate blood pressure control, whereas the other regressed completely following rigorous systemic hypertension management.

**Conclusions:**

Multiple RAMs within the same eye can follow distinctly divergent natural courses. In this single case, early blood pressure control appeared to be an important contributing factor to the divergent lesion outcomes, although the contribution of spontaneous regression and other confounding factors cannot be excluded. Multimodal imaging is indispensable for differentiating NVCM from vasogenic macular edema, guiding individualized conservative management, and preventing unnecessary interventions.

## 1 Introduction

First described by Robertson in 1973, retinal arterial macroaneurysm (RAM) is an uncommon acquired retinal vascular disorder characterized by saccular or fusiform dilation of major retinal arteries, predominantly occurring at arteriovenous crossings along the first- to third-order branches of the central retinal artery (1). The estimated incidence of RAM is approximately 1 in 9000 (2), with a strong demographic predilection for older adults (91% aged >60 years) and women (73%) (3). The pathogenesis of RAM is closely linked to systemic cardiovascular comorbidities, most notably hypertension, arteriosclerosis, and hyperlipidemia (4). Clinically, the condition is overwhelmingly unilateral in approximately 90% of cases (5, 6), with 89% of patients presenting with a solitary RAM lesion (3).

The clinical presentation of RAM is dictated largely by its anatomical proximity to the macula. While extramacular lesions are typically asymptomatic, RAM rupture can precipitate multilayered retinal or vitreous hemorrhage, resulting in profound and potentially irreversible vision loss. Although various therapeutic modalities are currently employed in clinical practice—including laser photocoagulation, intravitreal anti-vascular endothelial growth factor (VEGF) agents, and vitreoretinal surgery—the rarity of RAM and the marked heterogeneity of its natural history preclude the establishment of standardized guidelines or consensus regarding optimal treatment timing and strategies (7).

Herein, we report a patient with two distinct RAMs in a single eye, complicated by rare nonvascular cystoid maculopathy (NVCM), which exhibited markedly divergent clinical trajectories over a 5-year follow-up period: one underwent chronic fibrotic involution, whereas the other regressed completely.

## 2 Case reports

### 2.1 Initial presentation and disease progression

A 65-year-old woman presented with a 1-day history of sudden-onset floaters and visual obscuration in her left eye. She denied any prior systemic diseases including hypertension, diabetes mellitus or hyperlipidemia.

On initial ophthalmic examination on September 5, 2020, best-corrected visual acuity (BCVA; decimal notation) was 0.8 in the right eye and 0.25 in the left eye. Optical coherence tomography (OCT) demonstrated an extensive subretinal hemorrhagic elevation involving the inferotemporal macula of the left eye, accompanied by fluid accumulation beneath the neurosensory retina (Figure 1). Based on these findings, a provisional diagnosis of retinal hemorrhage in the left eye was established, and oral iodized lecithin (3 mg three times daily) was initiated to promote hemorrhage resorption.

**Figure 1:**
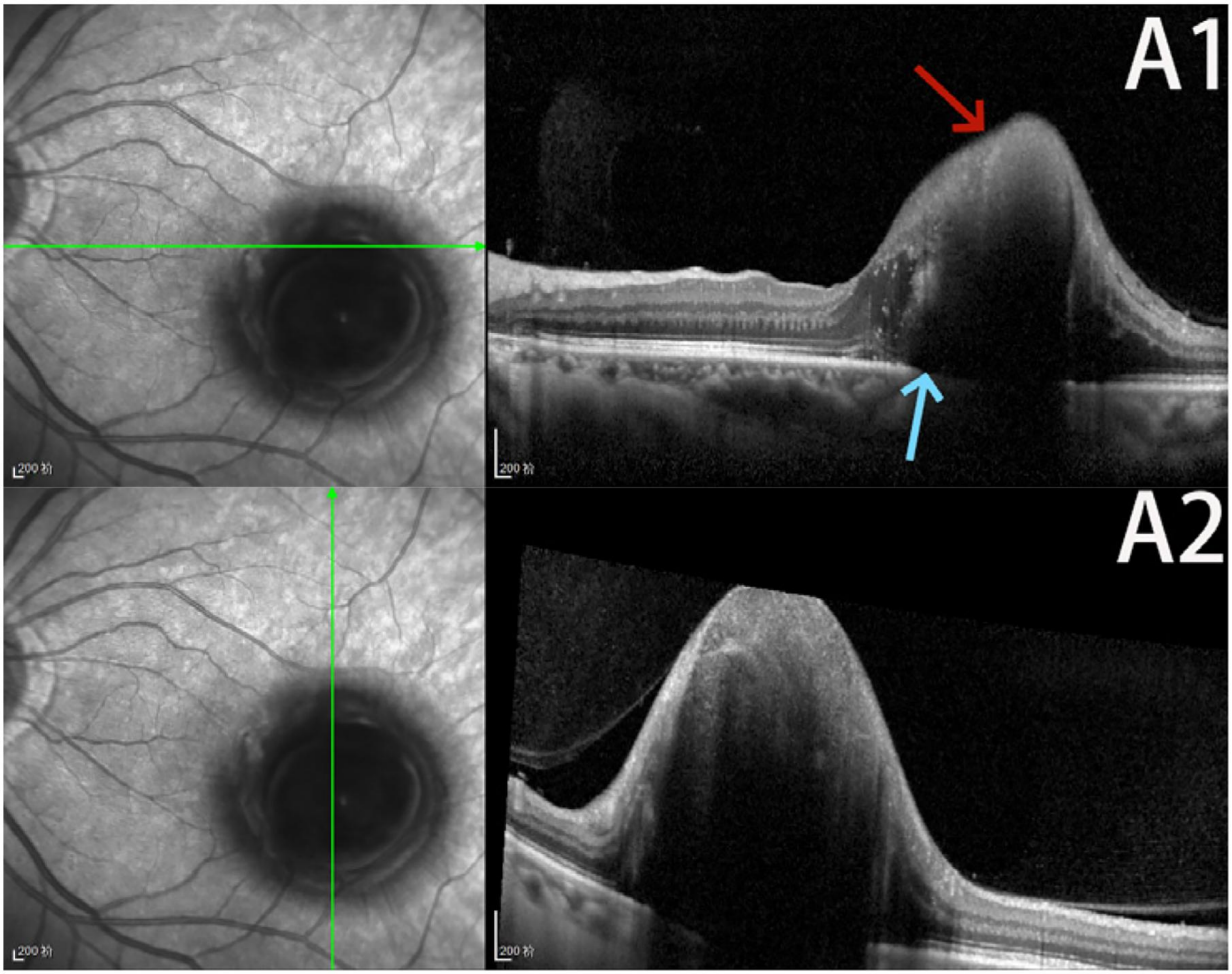
OCT demonstrates extensive neurosensory retinal elevation involving the inferotemporal macula (red arrow), with a hyporeflective subretinal fluid space beneath the elevated retina (blue arrow) (Figure **A1**). Longitudinal views of the lesion showed similar information (Figure **A2**).

At a follow-up visit on September 30, 2020, the patient reported progressive enlargement of the visual obscuration in her left eye since the day following her initial presentation. BCVA remained 0.8 in the right eye but had deteriorated to hand motions at 60 cm in the left eye. Blood pressure (BP) was markedly elevated at 187/104 mmHg, although she denied symptoms suggestive of hypertensive emergency, including headache, dizziness, nausea, or chest pain. Slit-lamp biomicroscopic examination with a fundus lens disclosed mild vitreous hemorrhage and expansion of the subretinal hemorrhage, while repeat OCT demonstrated progressive worsening of the neurosensory retinal elevation with accumulating subretinal blood components in the left eye (Figure 2). Given the patient's refusal of intravitreal anti-VEGF therapy, systemic antihypertensive treatment was initiated with oral nifedipine controlled-release tablets (30 mg twice daily) and metoprolol succinate sustained-release tablets (47.5 mg once daily), and she was instructed to maintain strict bed rest to minimize the risk of further hemorrhage.

**Figure 2:**
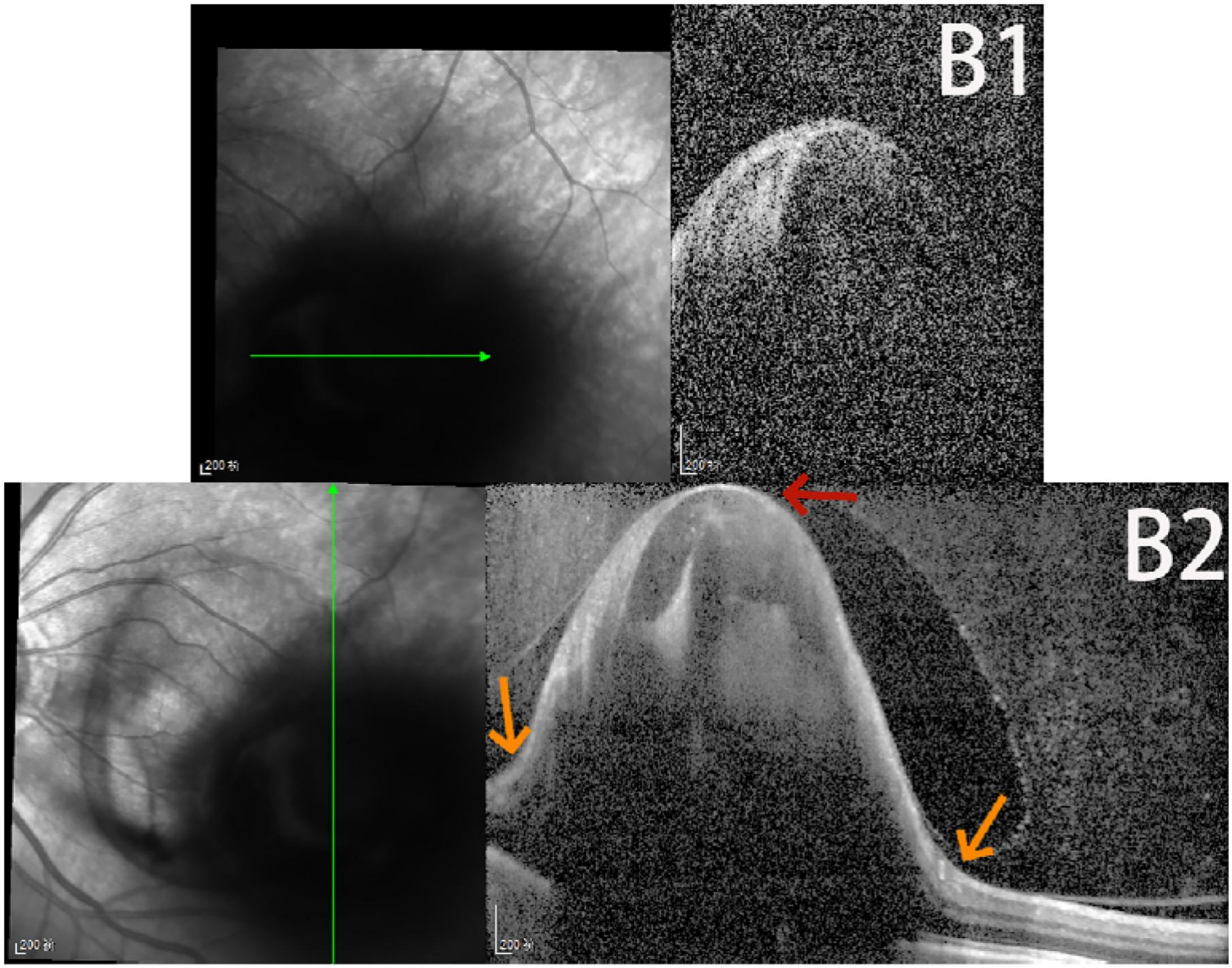
OCT reveals progressive enlargement (orange arrow) and increased height (red arrow) of the neurosensory retinal elevation in the inferotemporal macula compared with the prior scan, with numerous punctate hyporeflective to isoreflective signals within the vitreous cavity (Figure **B2**). Transverse views of the lesion showed similar information (Figure **B1**).

At the third follow-up visit on October 2, 2020, the patient reported profound visual deterioration in her left eye. Best-corrected visual acuity remained 0.8 in the right eye, whereas the left eye had deteriorated to light perception. BP was 163/99 mmHg. B-scan ultrasonography revealed extensive vitreous hemorrhage in the left eye, with concomitant elevation of both the neurosensory retina and the retinal pigment epithelium (Figure 3). OCT could not be performed at this visit because of dense vitreous hemorrhage. Pars plana vitrectomy (PPV) was recommended following further optimization of BP control, to which the patient consented. The antihypertensive regimen was subsequently intensified to nifedipine controlled-release tablets (60 mg twice daily) and metoprolol succinate sustained-release tablets (95 mg once daily), and strict bed rest was reiterated.

**Figure 3:**
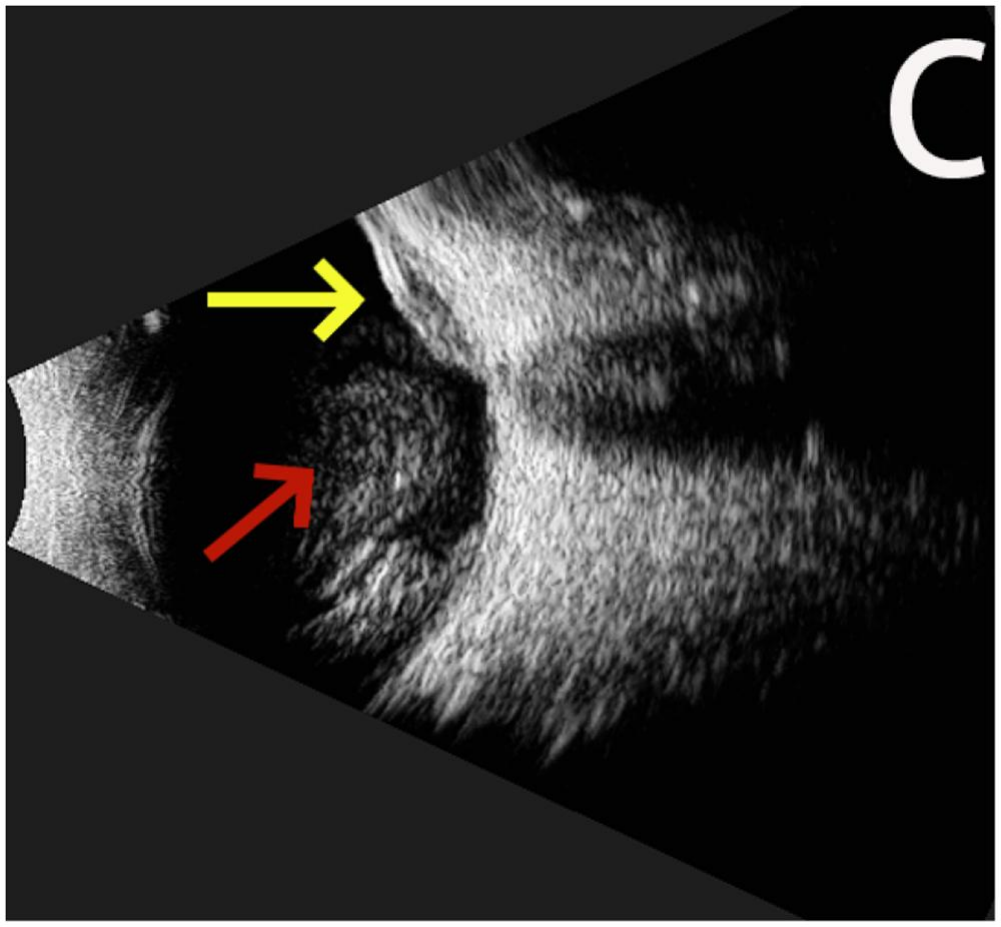
B-scan ultrasonography demonstrates extensive vitreous hemorrhage (red arrow) and a hyperreflective band representing elevated neurosensory retina anterior to the posterior vitreous face, with a hyporeflective subretinal space between the retina and retinal pigment epithelium (yellow arrow) (Figure **C**).

### 2.2 Surgical intervention and postoperative complication management

Fourth visit (November 5, 2020): 27 days postoperatively, the patient returned for follow-up. She reported that on October 9, 2020, due to persistent visual impairment, she had undergone left eye PPV combined with internal limiting membrane peeling, intravitreal recombinant tissue plasminogen activator (rt-PA) thrombolysis, fluid-air exchange, and silicone oil tamponade at a tertiary ophthalmic center. Postoperative BCVA in the left eye was 0.1. At the current visit, BCVA was 0.8 in the right eye and hand motion at 20 cm in the left eye. Intraocular pressure (IOP) was 17 mmHg in the right eye and 46 mmHg in the left eye. BP was 127/80 mmHg. Slit-lamp examination of the left eye revealed ciliary injection, anterior chamber hemorrhage with a 3-mm hyphema level, and a sluggish pupillary light reflex. Because of severe opacity of the ocular media, fundus OCT imaging could not be obtained; anterior segment OCT was therefore performed as an alternative means of documentation (Figure 4). Episodic intraocular hypertension secondary to hyphema with anterior chamber angle obstruction was diagnosed. Given the acute onset and the absence of documented optic nerve head cupping or visual field defects at this stage, this was regarded as transient secondary ocular hypertension rather than established glaucomatous optic neuropathy. The patient was initiated on 20% mannitol (250 mL intravenous infusion) and acetazolamide tablets (50 mg twice daily) for intraocular pressure reduction.

**Figure 4:**
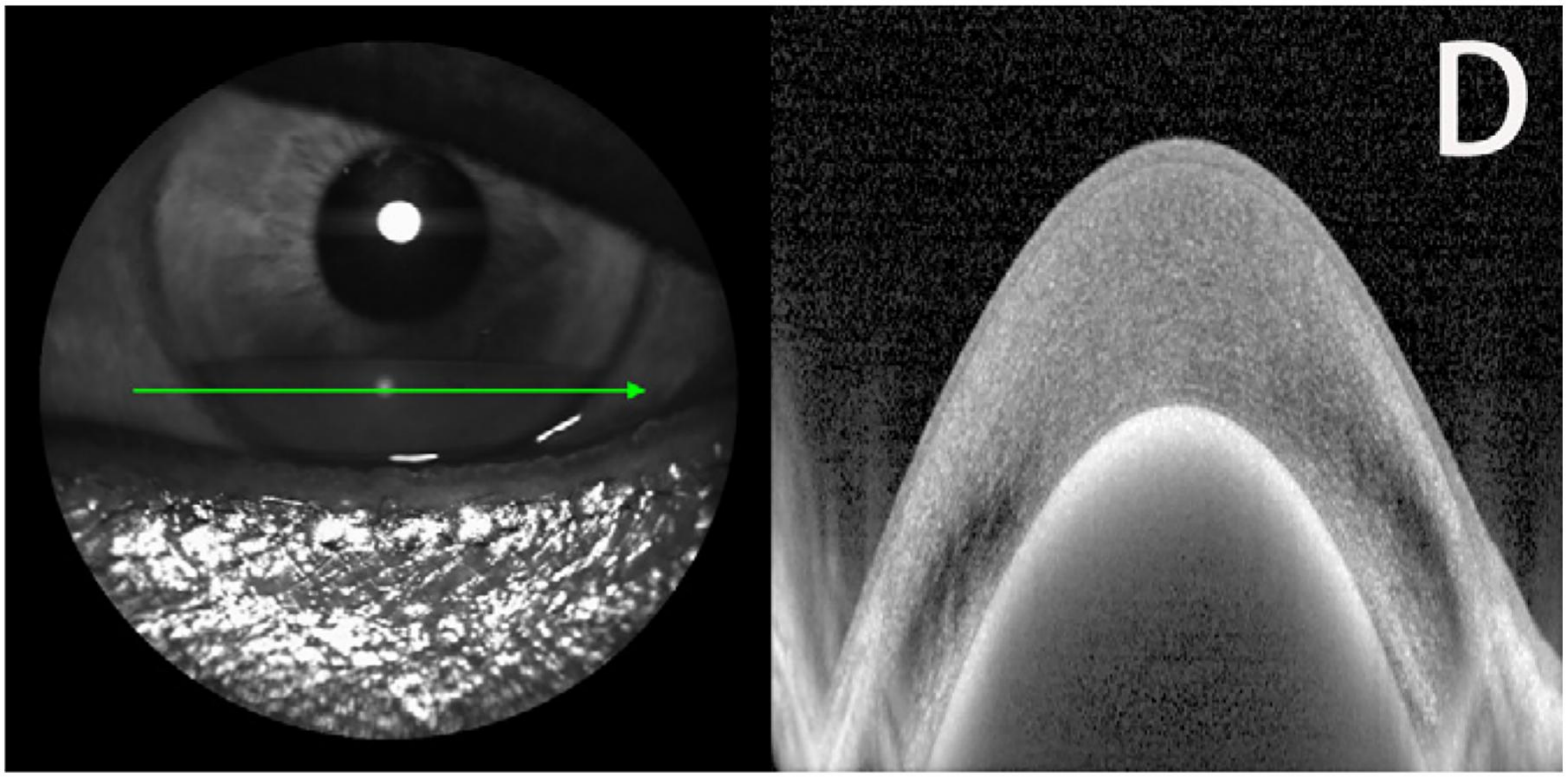
Anterior segment OCT image shows a 3-mm hyphema with a visible blood fluid level (Figure **D**).

Fifth visit (November 15, 2020): The patient's BCVA was 0.8 in the right eye and hand motion at 10 cm in the left eye. Intraocular pressure was 17 mmHg in the right eye and 34 mmHg in the left eye. BP was 127/83 mmHg. Anterior chamber irrigation with balanced salt solution was performed in the left eye. Postoperatively, the hyphema resolved, the anterior chamber angle reopened, and IOP normalized.

### 2.3 Confirmation of NVCM secondary to two suspected RAMs in the left eye

Sixth visit (January 13, 2021): The patient reported that she had undergone silicone oil removal surgery at another hospital and experienced improved vision. BCVA was 1.0 in the right eye and 0.02 in the left eye. Intraocular pressure was 13 mmHg bilaterally. BP was 125/85 mmHg. OCT revealed a large area of hyperreflective subretinal fluid beneath the neurosensory retina at the lesion site, and infrared fundus imaging demonstrated a prominent elevation at the lesion. Most of the lesion exhibited retinal pigment epithelium background reflectivity, with focal fresh hemorrhage observed. The peripheral area of the lesion showed cystoid macular changes in the neurosensory retina and subretinal fluid accumulation (Figure 5). The following day, combined fluorescein fundus angiography (FFA) and indocyanine green angiography (ICGA) were performed. In the late phase of FFA, a well-demarcated hypofluorescent lesion with mild peripheral fluorescence leakage was identified at one of the hemorrhagic sites, and the draining veins appeared tortuous. In the late phase of ICGA, the same lesion demonstrated hypofluorescence with mild peripheral leakage, and a second hemorrhagic site without leakage was observed as a hypofluorescent area (Figure 6). Based on these findings, the diagnosis of NVCM secondary to suspected RAM in the left eye was confirmed. Given that neither lesion exhibited active leakage and the patient's BP was well controlled, a conservative observation strategy was adopted without retinal laser photocoagulation.

**Figure 5:**
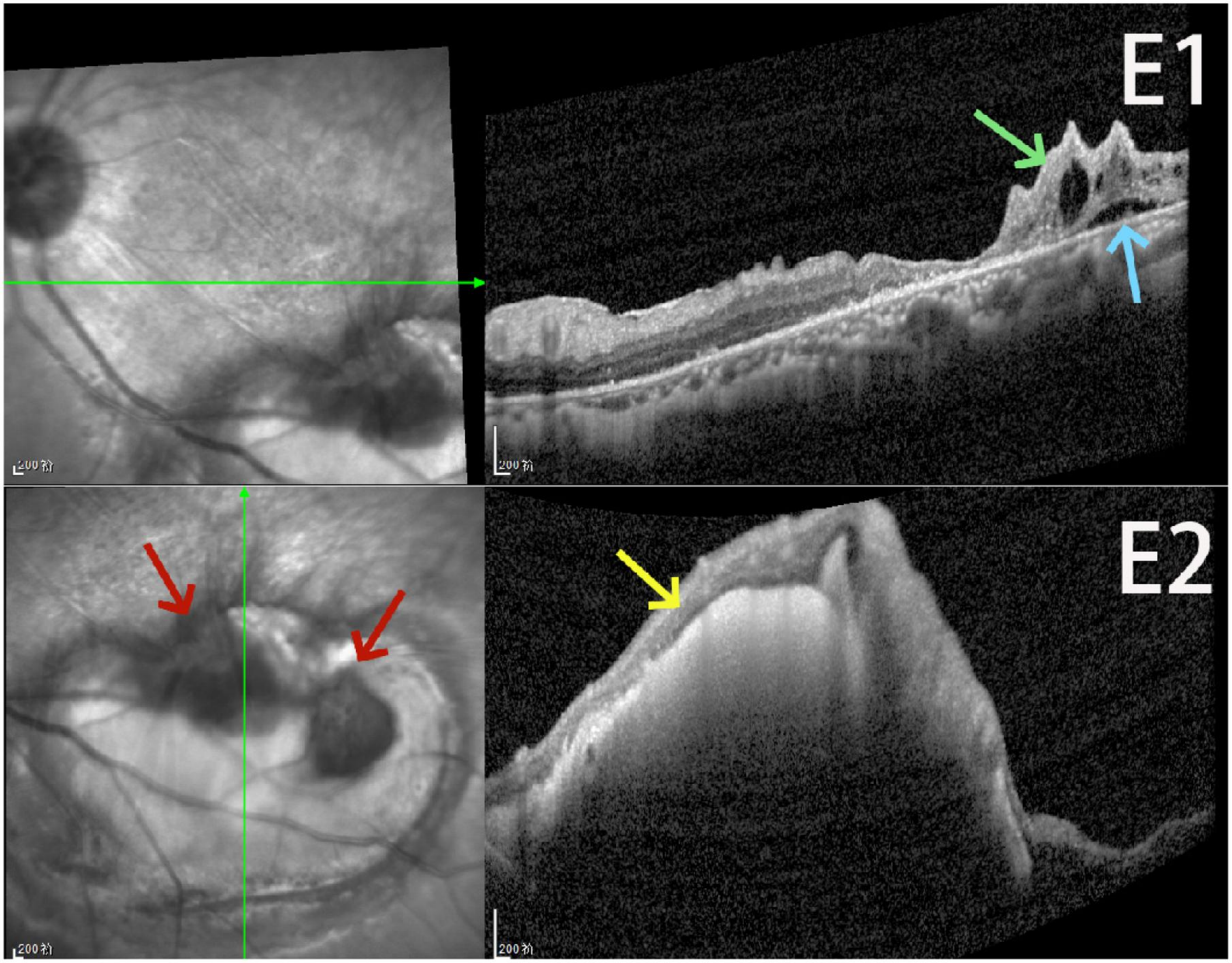
OCT demonstrates intraretinal cystoid spaces (green arrow) and hyporeflective subretinal fluid beneath the neurosensory retina in the foveal region (blue arrow) (figure **E1**). At the lesion site, a large area of hyperreflective subretinal material causes marked elevation of the neurosensory retina (yellow arrow). Corresponding infrared reflectance imaging reveals preserved retinal pigment epithelium background reflectivity overlying the elevated lesion, with focal fresh hemorrhages (red arrow) (Figure **E2**).

**Figure 6:**
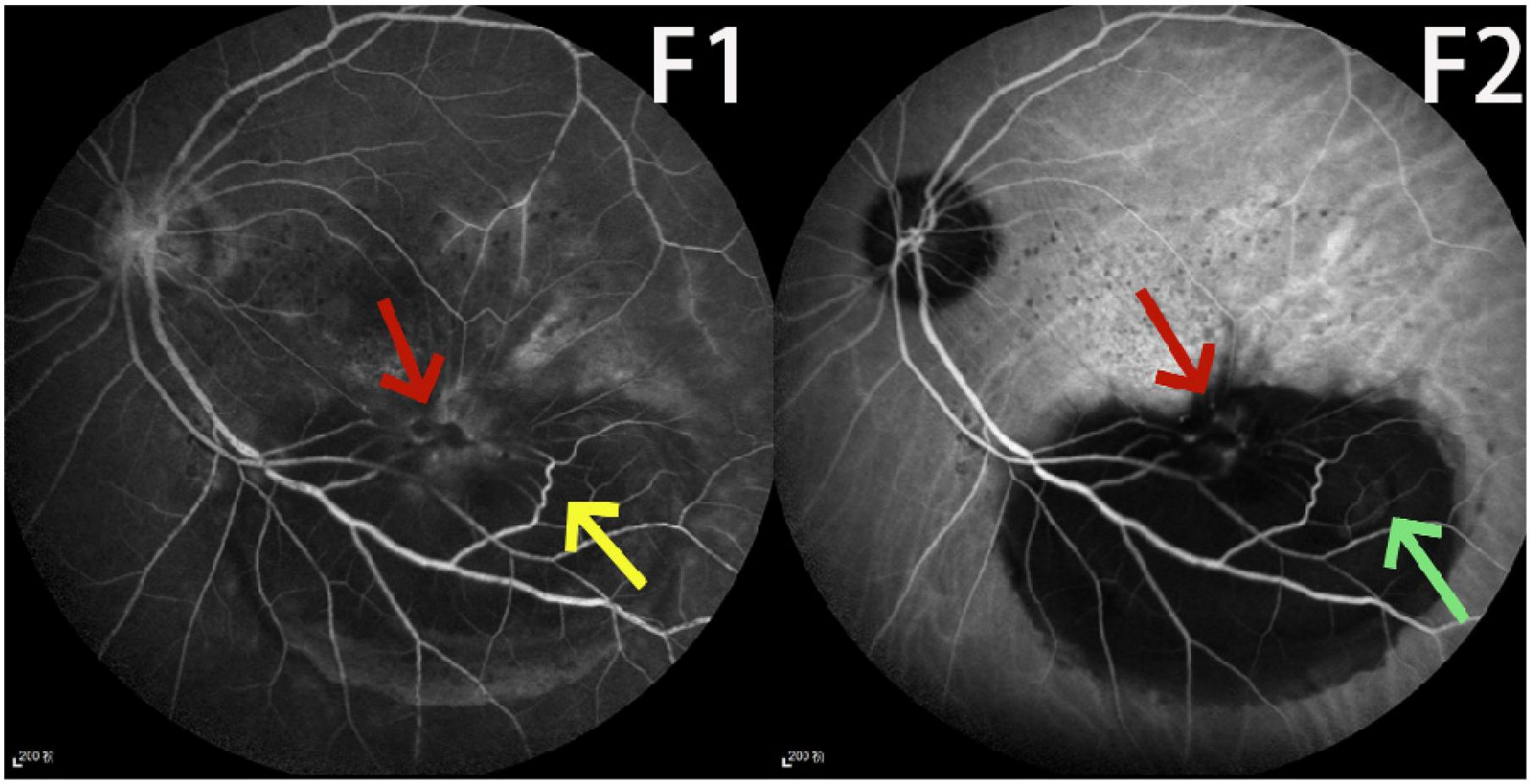
In the late phase of FFA, a well-demarcated hypofluorescent lesion was observed at one hemorrhagic site, accompanied by mild peripheral fluorescence leakage (red arrow) and tortuous draining veins (yellow arrow) (figure **F1**). In the late phase of ICGA, the same lesion demonstrated complete fluorescence blockage with mild peripheral leakage (red arrow), and a second hypofluorescent lesion without leakage was identified at another hemorrhagic site (green arrow) (Figure **F2**).

### 2.4 Long-term follow-up: distinctive outcomes of the two suspected RAMs

Eighth visit (October 28, 2021) (Figure 7H); ninth visit (March 9, 2022) (Figure 7I); tenth visit (August 27, 2022) (Figure 7J); eleventh visit (July 20, 2023) (Figure 7K); twelfth visit (August 27, 2024) (Figure 7L); thirteenth visit (August 11, 2025) (Figure 7M). During the follow-up period, BCVA in the left eye remained between 0.08 and 0.12. IOP and BP remained stable. OCT revealed that the *de novo* lesion was no longer identifiable, with the surrounding retina remaining flat. The area of retinal elevation surrounding the initial lesion had significantly decreased compared to the seventh visit. The height of the retinal elevation exhibited a gradual declining trend over the four-year period, and the peripheral structures at the apex demonstrated marked hyperreflectivity, accompanied by persistent intraretinal cystoid spaces around the lesion. B-scan ultrasonography consistently demonstrated a protruding lesion extending toward the vitreous cavity from the posterior globe wall. The main information of this patient's previous visits has been summarized in Table 1.

**Figure 7:**
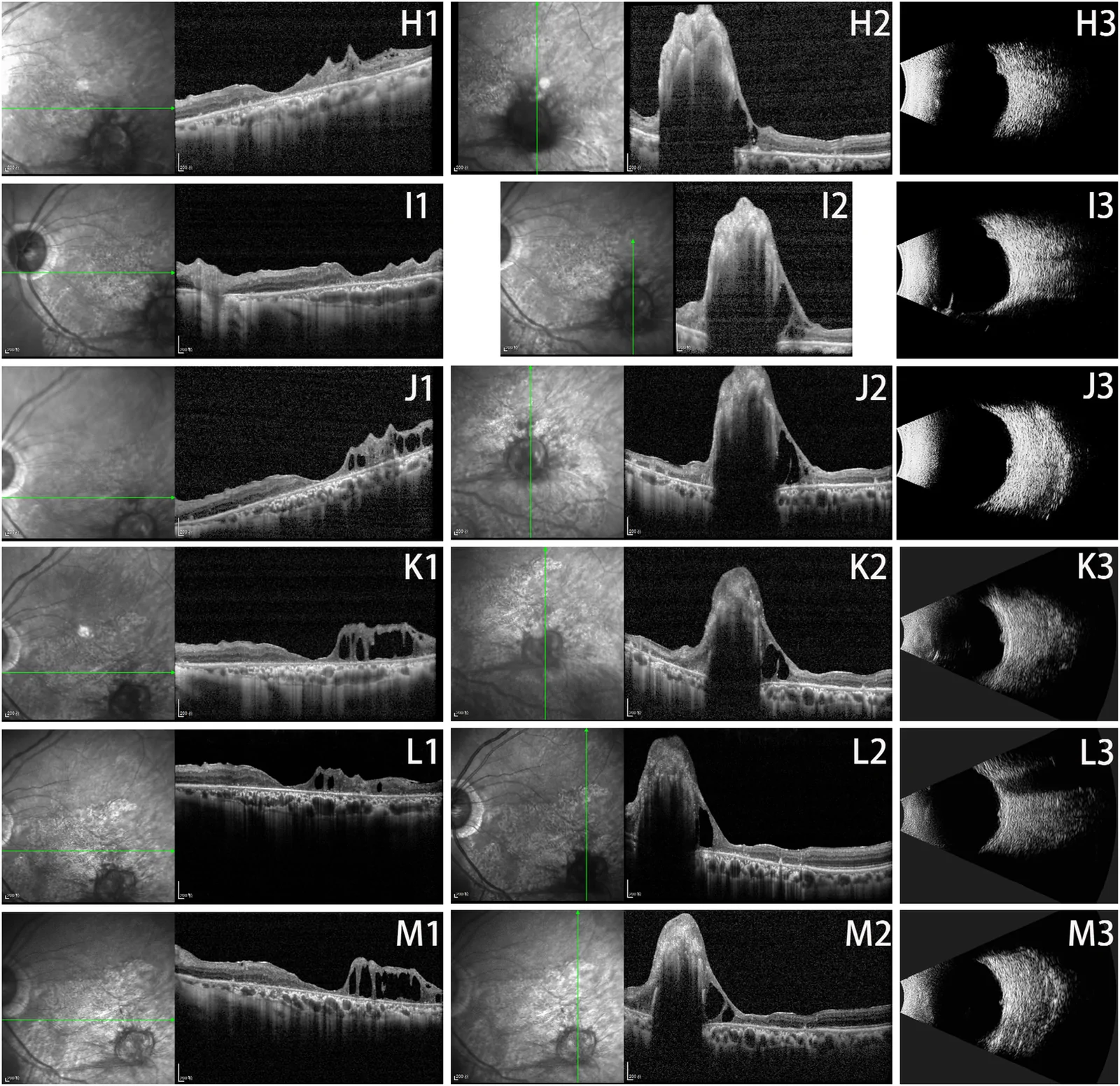
**(H–M)** serial OCT and B-scan ultrasonography from the eighth to thirteenth visits demonstrated progressive reduction in the extent and height of retinal elevation surrounding the first lesion compared to the seventh visit. The perilesional structures at the apex exhibited marked hyperreflectivity, with persistent intraretinal cystoid spaces. B-scan ultrasonography consistently revealed a protruding lesion extending into the vitreous cavity from the posterior globe wall.

**Table 1:** Chronological summary of the clinical course.

Visit (date)	BCVA (OD/OS)	BP (mmHg)	Key imaging findings (Figure)	Interventions and outcomes
Initial visit (Sep 5, 2020)	0.8/0.25	–	OCT: extensive subretinal hemorrhagic elevation, inferotemporal macula (Figure 1)	Provisional diagnosis of retinal hemorrhage; oral iodized lecithin
Second visit (Sep 30, 2020)	0.8/HM 60 cm	187/104	OCT: progressive elevation with subretinal blood; mild vitreous hemorrhage (Figure 2)	Nifedipine + metoprolol initiated; strict bed rest
Third visit (Oct 2, 2020)	0.8/LP	163/99	B-scan: extensive vitreous hemorrhage with retinal elevation (Figure 3)	Antihypertensives intensified; PPV planned
Fourth visit (Nov 5, 2020)	0.8/HM 20 cm	127/80	Hyphema (3 mm) with angle obstruction (Figure 4)	PPV + rt-PA + ILM peeling + silicone oil (Oct 9, 2020); mannitol, acetazolamide
Fifth visit (Nov 15, 2020)	0.8/HM 10 cm	127/83	Hyphema persisting	Anterior chamber irrigation; hyphema resolved, IOP normalized
Sixth visit (Jan 13, 2021)	1.0/0.02	125/85	OCT/IR: two hemorrhagic foci with cystoid changes; late-phase FFA/ICGA: hypofluorescence with perilesional staining (Figures 5,6)	Silicone oil removal (elsewhere); conservative observation adopted
Eighth–thirteenth visits (Oct 2021–Aug 2025)	−/0.08–0.12	Stable	*De novo* lesion resolved; initial lesion fibrotic with persistent cystoid spaces (Figure 7)	Conservative observation

## 3 Discussion

Most patients with RAM present with acute vision loss secondary to RAM rupture. Fundus examination typically reveals multilayered hemorrhages involving the subretinal, intraretinal, and preretinal spaces, as well as the vitreous cavity. This presentation closely mimics various hemorrhagic retinopathies, resulting in a misdiagnosis rate as high as 75% (8). In the present case, extensive intraretinal hemorrhage at initial presentation obscured the suspected primary RAM lesion, precluding a definitive diagnosis of RAM by FFA/ICGA, the diagnostic gold standard. Treatment was therefore symptomatic and directed at the nonspecific retinal hemorrhage.

The pathogenesis of RAM is generally attributed to arterial wall damage and impaired perfusion caused by local embolic events affecting weakened vessel walls. This process subsequently upregulates VEGF expression, thereby increasing vascular permeability and promoting vascular dilation (9). Hypertension is the most significant systemic risk factor for RAM, with 51% to 75% of affected patients presenting with concurrent hypertension (3, 10, 11). During the patient's second visit, intraretinal hemorrhage had progressed, resulting in further elevation of the retinal lesion as demonstrated on OCT. When an extensive hemorrhagic macular lesion is identified, the main differential diagnoses include polypoidal choroidal vasculopathy, Valsalva retinopathy, exudative age-related macular degeneration, retinal vein occlusion, and diabetic retinopathy. In the present case, the patient initially denied any Valsalva maneuver, trauma, or use of anticoagulant/antiplatelet agents. The lesion was located above the retinal pigment epithelium, and no drusen, flame-shaped retinal hemorrhages, or irregular retinal hemorrhages were observed. Based on these clinical features, other diagnoses were excluded, and a presumptive diagnosis of RAM was made. Although numerous studies have reported the efficacy of anti-VEGF therapy for RAM treatment (9, 10, 12–15), this patient declined anti-VEGF intervention and opted for conservative management. Consequently, the patient was instructed to maintain strict BP control and limit physical activity to prevent further hemorrhagic complications.

Ruptured RAM may result in simultaneous hemorrhage into multiple compartments, including the vitreous cavity, preretinal space, intraretinal layers, and subretinal space. Among these, subretinal hemorrhage carries the poorest prognosis and, if left untreated, may lead to severe vision loss (16). Animal studies have demonstrated that subretinal hemorrhage can cause significant photoreceptor and outer nuclear layer damage within 24 h. If untreated for 7 to 14 days, it results in irreversible damage to photoreceptors and the retinal pigment epithelium, leading to retinal atrophy and necrosis (17, 18). This pathological disruption of retinal integrity may permit red blood cell debris to traverse the compromised retina, thereby causing secondary vitreous hemorrhage in some patients (19, 20). When vitreous hemorrhage is concomitant, conservative management often fails to adequately promote intraocular blood resorption. Clinically, PPV is frequently employed to clear vitreous hemorrhage, restore vitreous clarity, and relieve retinal traction. At the third visit, the patient presented with extensive vitreous hemorrhage. B-scan ultrasonography revealed a large area of retinal elevation with thickening, strongly suggestive of concurrent subretinal hemorrhage. Therefore, to preserve the patient's visual function, PPV was recommended immediately following BP stabilization to restore optical media clarity and address potential subretinal hemorrhage.

Recurrent hemorrhage is a common postoperative complication following PPV, typically occurring in the early postoperative period. By the fourth visit, the patient had undergone surgical clearance of vitreous and subretinal hemorrhage. Despite adequate BP control, recurrent retinal bleeding occurred. The hemorrhage extended from the vitreous cavity through the pupil into the anterior chamber, resulting in persistent IOP elevation secondary to hyphema and angle obstruction. When conventional measures failed to promote blood resorption and control IOP, anterior chamber irrigation was performed at the fifth visit to prevent corneal blood staining. Given that the patient's BP remained well controlled, no adjustments were made to the systemic antihypertensive regimen following the procedure.

The current primary treatment for RAM involves direct or circumferential laser photocoagulation around the RAM, utilizing subthreshold or threshold lasers, including xenon arc, argon, and yellow dye lasers (21–23) This approach promotes intra-aneurysmal thrombosis, induces retinal vessel contraction, and stimulates vessel wall fibrosis, thereby facilitating RAM involution and hemorrhage resorption. However, in clinical practice, laser monotherapy demonstrates relatively slow resolution of macular edema and exudation (24), and carries risks including RAM rupture with hemorrhage, distal vascular occlusion, and visual field defects (25). Some studies have even reported visual acuity deterioration following laser treatment (26). In the present case, at the sixth visit, OCT revealed two distinct hemorrhagic foci. Subsequent FFA combined with ICGA demonstrated that the suspected initial RAM exhibited hypofluorescence with mild perifocal leakage in the late phase, while the suspected *de novo* RAM displayed faint hyperfluorescent outlining without leakage in the late-phase ICGA. We believe that this mild peripheral leakage represented reactive fluorescein leakage resulting from the abnormal retinal architecture caused by the large intraretinal blood clot, rather than active cystoid leakage attributable to neovascularization, because the site of leakage on FFA was spatially distinct from the intraretinal cystoid spaces observed on OCT. Based on imaging evidence indicating stability of both RAMs without active leakage, we adopted a conservative approach and withheld laser photocoagulation to avoid overtreatment. This decision was validated during the subsequent 4-year follow-up period.

The primary clinical significance of this case lies in demonstrating the markedly divergent natural histories of two suspected sequential RAMs in a single eye. Literature reports indicate that RAM generally carries a favorable prognosis with a tendency toward spontaneous regression. Most RAM patients experience visual improvement even without intervention (27), and some patients with concurrent hypertension achieve complete recovery through antihypertensive therapy alone (28, 29). Notably, certain patients have demonstrated substantial visual recovery without any treatment (30–32) During the 5-year follow-up period, the inital and *de novo* lesions in this case exhibited a striking contrast of “persistence versus resolution”: the inital lesion, attributable to uncontrolled BP at disease onset, extensive hemorrhage, and subretinal involvement, ultimately evolved into an elevated retinal scar accompanied by outer retinal atrophy in the surrounding area. Conversely, the *de novo* lesion, detected relatively early with limited hemorrhage and managed with strict BP control, had completely resolved by the eighth visit. This contrast compellingly underscores the critical importance of early BP management and timely intervention in determining long-term RAM prognosis. However, the two lesions also differed in hemorrhage severity, timing of detection, treatment history, and probably disease stage, and spontaneous regression of RAM is well recognised. Blood pressure control should therefore be regarded as an important possible contributing factor to the divergent outcomes rather than a decisive prognostic determinant in this single case, and the complete resolution of the *de novo* lesion may partly reflect its natural tendency towards spontaneous involution.

Furthermore, this case represents the first reported occurrence of NVCM secondary to suspected RAM. The pathophysiology of NVCM involves various pathological factors that compromise retinal cellular adhesion or impair the functions of retinal pigment epithelium and Müller cells in maintaining retinal dehydration, resulting in the formation of intraretinal cystic spaces (33). FFA is essential for differentiating NVCM from cystoid macular edema caused by blood-retinal barrier disruption, as fluorescein does not leak into the cystic spaces characteristic of NVCM. NVCM is commonly associated with non-exudative age-related macular degeneration (34), type 2 macular telangiectasia (35), hereditary retinal dystrophies [including X-linked retinoschisis [36], enhanced S-cone syndrome (37), and retinitis pigmentosa (38)], acquired RPE dysfunction [such as central serous chorioretinopathy (39) and paraneoplastic syndromes (40)], optic nerve disorders [including primary open-angle glaucoma (41), multiple sclerosis (42), and optic neuritis (43)], drug-induced retinopathies [associated with tamoxifen (44), taxanes (45), and niacin (46)], and vitreomacular interface disorders [including vitreomacular traction (47), epiretinal membrane (48), and myopic foveoschisis (49)]. During the more than 4-year follow-up period from the sixth to thirteenth visits, although cystoid changes persisted around the lesion, the patient's visual acuity and lesion morphology remained stable. FFA at the sixth visit revealed no apparent leakage or areas of capillary non-perfusion. Therefore, we conclude that this patient developed chronic NVCM secondary to suspected RAM, although it is possible that the initial NVCM was caused by mechanical traction related to surgery. The underlying mechanism likely involves tractional forces from the suspected cicatricial RAM on the surrounding retina, thereby compromising Müller cell function and structural integrity.

## 4 Conclusion

This case demonstrates the markedly divergent outcomes of unilateral two suspected RAMs over a long-term follow-up period. In this single case, blood pressure control appeared to be an important contributing factor to the divergent lesion trajectories, although spontaneous regression and other confounding factors cannot be excluded. It also represents the first report of the rare imaging features of NVCM secondary to RAM. The contrasting evolutionary trajectories of the initial and the *de novo* lesions—characterized by “persistence versus resolution”—underscore the potential importance of early blood pressure control in long-term RAM prognosis. Furthermore, multimodal imaging, particularly FFA combined with ICGA, plays an indispensable role in precisely assessing lesion activity, differentiating NVCM from cystoid macular edema, and guiding individualized decision-making to avoid overtreatment. This report provides valuable long-term evidence-based insights into the natural course of RAM and the clinical management of its complex complications.

## 5 Limitation

Our study has several limitations that warrant acknowledgment. As a single retrospective case report of two suspected unilateral RAMs complicated by secondary NVCM—an exceptionally rare phenotype—our findings carry limited generalizability, and the absence of a matched control group precludes definitive causal inferences linking blood pressure management to the divergent lesion trajectories, as well as quantitative comparisons between conservative care and active interventions including laser photocoagulation and anti-VEGF therapy. Furthermore, color fundus photography and angiography were not available during the acute phase, and because the ocular media did not clear sufficiently to permit reliable FFA and ICGA until 4 months after symptom onset, the early- and mid-phase frames could not demonstrate the vascular origin and arterial continuity of the RAM, which had already assumed an end-stage appearance by the time of imaging. Only the late-phase frames provided incomplete evidence in support of our diagnosis; accordingly, the diagnosis of RAM in this patient could only remain presumptive. Moreover, the non-standardized treatment regimen, driven in part by the patient's declination of anti-VEGF therapy, introduces substantial confounding; with multiple sequential and concurrent interventions, the independent therapeutic contribution of any single modality cannot be reliably isolated, and the observed lesion regression may partially reflect the well-documented natural tendency of RAM toward spontaneous involution rather than a direct treatment effect. Finally, our proposed pathophysiological mechanism for RAM-associated NVCM, inferred exclusively from multimodal imaging features, remains hypothetical in the absence of histopathological confirmation or complementary functional testing such as microperimetry. Notwithstanding these limitations, this 5-year longitudinal case provides valuable real-world insights into the heterogeneous natural history of RAM and its uncommon complications.

## Data Availability

The original contributions presented in the study are included in the article/Supplementary Material, further inquiries can be directed to the corresponding author.
